# Data for Improvement and Clinical Excellence: a report of an interrupted time series trial of feedback in home care

**DOI:** 10.1186/s13012-017-0600-1

**Published:** 2017-05-18

**Authors:** Kimberly D. Fraser, Anne E. Sales, Melba Andrea B. Baylon, Corinne Schalm, John J. Miklavcic

**Affiliations:** 1 Faculty of Nursing, Level 3 Edmonton Clinic Health Academy, University of Alberta, 11405 – 87 Ave NW, Edmonton, AB T6G 1C9 Canada; 2 Centre for Clinical Management Research, VA Ann Arbor Healthcare System, 2215 Fuller Road, PO Box 130170, Ann Arbor, MI 48113 USA; 3 Alberta Health and Wellness, 10025 Jasper Ave NW, Edmonton, AB T5J 1S6 Canada

**Keywords:** Audit with feedback intervention, Interrupted time series, Home care, Quality improvement, Process evaluation

## Abstract

**Background:**

There is substantial evidence about the effectiveness of audit with feedback, but none that we know have been conducted in home care settings. The primary purpose of the Data for Improvement and Clinical Excellence – Home Care (DICE-HC) project was to evaluate the effects of an audit and feedback delivered to care providers on home care client outcomes. The objective of this paper is to report the effects of feedback on four specific quality indicators: pain, falls, delirium, and hospital visits.

**Methods:**

A 10-month audit with feedback intervention study was conducted with care providers in seven home care offices in Alberta, Canada, which involved delivery of four quarterly feedback reports consisting of data derived from the Resident Assessment Instrument – Home Care (RAI-HC). The primary evaluation employed an interrupted time series design using segmented regression analysis to assess the effects of feedback reporting on the four quality indicators: pain, falls, delirium, and hospitalization. Changes in level and trend of the quality indicators were measured before, during, and after the implementation of feedback reports. Pressure ulcer reporting was analyzed as a comparator condition not included in the feedback report. Care providers were surveyed on responses to feedback reporting which informed a process evaluation.

**Results:**

At initiation of feedback report implementation, the percentage of clients reporting pain and falls significantly increased. Though the percentage of clients reporting pain and falls tended to increase and reporting of delirium and hospital visits tended to decrease relative to the pre-intervention period, there was no significant effect of feedback reporting on quality indicators during the 10-month intervention. The percentage of clients reporting falls, delirium, and hospital visits significantly increased in the 6-month period following feedback reporting relative to the intervention period. About 50% of the care providers that read and understand the feedback reports found the reports useful to make changes to the way clients are cared for.

**Conclusions:**

Routinely collected data used over time for feedback is feasible in home care settings. A high proportion of care providers find feedback reports useful for informing how they care for clients. Since reporting on the frequency of quality indicators increased in the post-intervention period, this study suggests that ongoing use of audit with feedback to enhance health outcomes in home care may promote improved reporting on standardized instruments.

**Electronic supplementary material:**

The online version of this article (doi:10.1186/s13012-017-0600-1) contains supplementary material, which is available to authorized users.

## Background

Home care consists of an array of services designed to meet individual client needs [1, 2]. Based on census data from 2003, it was estimated that 550,000 unique clients aged 65 and older received home care in Canada [3], and by 2006, this number grew to nearly one million home care clients at any given time [2]. Home care is increasing in both numbers and in level of client acuity [4, 5] across Canada and in most other developed countries. It is typically part of the broader continuing care sector, which also includes long-term care (facility living) and assisted or supportive living within the Canadian healthcare system. Home care is not included in the *Canada Health Act* as a necessary medical service and as such receives variable public funding across Canadian jurisdictions through provincial governments [6]. The objective of home care is to provide cost-effective ongoing, home-based care for clients of all age groups following hospital discharge, for palliation, aging in place, or for those that are cognitively and physically impaired [5]. Home care is provided based on assessed need. The assessment is done by case managers, most of whom are registered nurses, but may also be an allied health professional. Case managers have complex caseloads that vary depending on the type of client, for example, whether they are short-term post hospital care, pediatric, or long-term maintenance clients [7]. Caseloads are typically culturally diverse and include clients with a myriad of diagnoses. Following assessment, case managers care plan with the client and family, coordinate care and services required, monitor the client response to care, and ensure client safety among other duties [1, 6, 8].

Case managers in Canada are the gate keeper to home care. Care is delivered most frequently by healthcare aides, followed by licensed practical nurses, registered nurses, and allied health professionals. Direct care is either provided directly through a government home care program or more typically in many Canadian jurisdictions through a contracted services provider agency that works closely with the case manager from intake to discharge. In contractual arrangements, the case manager determines and allocates care while the service provider agency hires, supervises, and manages all aspects of the staffing component. The case manager determines the plan of care, and the service provider agency carries out the plan of care.

This project arm is part of a larger group of DICE studies [9–12] and focused on the home care setting specifically, as opposed to long-term care (LTC) or other healthcare settings. The LTC project was reported elsewhere [11].

This arm of the study focused on the feedback as a quality improvement practice in the home care setting [9]. In home care, quality improvement initiatives are increasing in number but still lag somewhat behind other continuing care sectors [13]. The availability of standardized tools such as the RAI-HC has made data more readily available. However, systems to use the data are often not imbedded in organizations which makes it challenging to compare outcomes so trials of quality improvement interventions are rarely conducted compared with other healthcare settings [13].

The evidence for specific interventions to support the implementation of evidence-based practices in healthcare settings is mixed at best. In addition to the findings of a recent systematic review by our team [13], a study by Markle-Reid et al. on nurse-led health promotion in southern Ontario illustrated that the use of standardized screening tools can improve the effectiveness of interventions in home care [14]. Another intervention that shows promise is the use of audit with feedback intervention (referred to as feedback intervention) [15, 16].

Feedback interventions have demonstrated a modest effect to promote desired behavioral changes among healthcare providers, across settings and provider types [15, 16]; and although proposed in 1989 as having positive implications for future nursing care [17], very few [18] feedback intervention studies have been conducted in home care settings. The lack of data on care processes and outcomes have posed a major barrier to using feedback interventions in home care settings. However, this has been somewhat improved with the implementation of the RAI-HC, a standardized assessment tool used in many jurisdictions around the world.

The probable mechanism by which feedback interventions has a main effect is in providing healthcare providers with information about their own performance [19–21]. It is thought that knowledge of one’s performance, particularly among those who have not received data-based feedback on their past performance, could contribute to an increase in motivation to change certain behaviors. Feedback reports have often been used in conjunction with other interventions to enhance a desired effect [15]. The purpose of this study was to adopt feedback reporting as a sole intervention in home care since providers often work alone in the community. As a result, there is less opportunity to interact with colleagues and other providers where they may receive feedback on performance. Feedback reporting constitutes a method by which home care providers can receive information on client outcomes directly related to provider care. The strengths of this feedback intervention lie in the widespread availability of data on outcomes using RAI-HC, the relative simplicity with which it is constructed, and that it does not require additional cost for audit data to construct feedback reports [22–24].

This paper reports the summative client outcomes of a feedback intervention that was delivered to all staff in seven home care settings and contains a process evaluation on uptake of feedback reporting. The primary hypothesis of this study was that a consistent, long duration, client-focused feedback intervention would improve client outcomes among four important quality indicators: pain, falls, delirium, and hospital visits.

## Methods

An interrupted time series design was employed to assess the overall effect of feedback reports in the home care setting. Based on the underlying conceptual model built on the theory of planned behavior [25], it was hypothesized that feedback reporting would influence intention to change behavior through multiple paths including attitudes and social norms [11]. We are unable to measure to provider behavior directly but report on elements of process evaluation. The full protocol for the Data for Improvement and Clinical Excellence project is available [9]. Methodology pertaining to the specific elements of DICE-HC is reported here.

### Settings and sample

Site selection was guided by the degree of RAI-HC implementation. Ultimately, seven home care offices in both rural and urban areas within Alberta participated in the study. All home care offices were part of Alberta Health Services which is the single health region in Alberta responsible for all health service delivery including home care. At the time of the study, there were five zones. These were South Zone covering areas south of the city of Calgary, Calgary Zone, Central Zone, Edmonton Zone, and North Zone covering all areas north of Edmonton. Participants included direct care staff: case managers, nurses, healthcare aides, and allied health professionals, as well as managers and home care professional practice leaders.

### The intervention: quality indicator feedback reports

The feedback reports were initially developed during a pilot study conducted in Edmonton area in 2007. The feedback reports were refined for home care using client quality indicator outcomes selected based on the RAI-HC. The outcomes of the RAI-HC indicate potential quality issues that may need further review and are used by care providers to monitor trends and improve care [26]. The quality indicators for this study were selected based on clinical importance. The quality indicators that were determined to be of most value to this project were determined using a consensus building process with a group of six decision-maker partners based on the following criteria:

- What is known from the literature about what is important
- The strategic initiatives in progress or likely to be initiated in the period of the project
- The comparability of the indicators (i.e.*,* were the indicators comparable/similar to those used in long-term care)
- What was useful to clinicians
- Where changes are likely to be observed

The decision-maker group consisted of home care clinical and administrative leaders who were part of the research team. They had a variety of clinical or administrative backgrounds including nursing, rehabilitation, and quality improvement. The consensus results showed that pain, falls, delirium, and hospital visits were the most important quality indicators to be included in the feedback report.

Pain was selected as the primary quality indicator for consistency throughout the continuing care sectors assessed. Pain was also the primary indicator assessed in the DICE-LTC project [10, 11] as provider awareness of client pain may prompt pain alleviation measures like a referral or prescription of medication. Pain was quantified by taking into account both the frequency and intensity with which the client complained or showed evidence of pain. A client was reported to be in pain if he or she had either (a) less than daily pain but it was horrible or excruciating; (b) one to more periods daily and it was moderate to severe; or (c) one to more periods daily and it was excruciating.

Falls were reported in terms of frequency, i.e.*,* the number of times a client has fallen in the last 90 days or since the last assessment if less than 90 days. In the present study, a client was recorded to have a fall if he or she had at least one fall during the current 90-day period.

A client reported delirium if either (a) there was sudden or new onset/change in mental function of the client over the last 7 days and/or (b) the client experienced agitation or disorientation in the last 90 days, or since the last assessment if less than 90 days, such that his or her safety is endangered or the client required protection by another person.

Clients reported a hospital visit if one occurred in the last 90 days or if since last assessment, they used at least one of the three services described: (a) admission to hospital with an overnight stay; (b) visit to emergency room without an overnight stay; (c) emergent care—including unscheduled nursing, physician, or therapeutic visits to the office or home.

Finally, the presence of pressure ulcer was assessed as a related quality indicator not included in the feedback report. Pressure ulcer was selected as a reference measure because it is sensitive to change that may occur independently of the response to feedback reporting. Clients were reported to have a pressure ulcer if they had at least stage 2 of this skin condition.

### Feedback report generation

Feedback reports specific to each home care office were then generated using the selected quality indicators based on data from the RAI-HC and included the percentages of clients with pain, falls, delirium, and hospital visits (Additional file 1). Client assessments using the RAI-HC are routinely conducted once annually or when there is a change in health status and were implemented during the study period under several circumstances: initial assessment, follow-up assessment, routine assessment at fixed intervals, review within the 30-day period prior to discharge from the home care program, review at return from hospital, and change in status. If a client had more than one assessment in a quarter, only the most recent assessment was recorded.

### Feedback report intervention

The first feedback report was based on the most recent data for one full year divided into four annual quarters, and in the succeeding reports, subsequent quarterly data were added with the previous first/earliest quarter being omitted. Feedback reports included data on 1 year of historical performance from the home care office staff to whom it was distributed and those data from other participating home care offices. Reports were distributed in June 2011, September 2011, December 2011, and March 2012. The intervention involved giving the feedback reports to the individual care provider either in person or via electronic distribution.

The in-person intervention was implemented in Edmonton, North, and Central Zone home care offices and electronically for South and Calgary Zones [9]. If it was not possible for staff to be in attendance in person, for example, if staff were out on home visits, on a different shift, or on a day off, reports were left in the office at an arranged location for pickup and review.

Electronic distribution was done using the Continuing Care Desktop that was developed by the Centre for Health Evidence in collaboration with Alberta Health and Wellness. All employees in continuing care in Alberta have access to the Continuing Care Desktop, and employee access is managed by each home care office. Site leaders were notified by email when the reports became available on the Continuing Care Desktop.

### Response to feedback reporting: Process evaluation

In addition to the feedback intervention, data on intention to change behavior based on feedback were also obtained. We provide brief description of the methods for the process evaluation here as the detailed protocol was published previously [9]. A sample survey is provided in the protocol paper [11]. One week after feedback reports were distributed, employees were asked to complete a survey electronically on the Continuing Care Desktop. The survey includes questions on demographics and an assessment of employee response to the feedback report. Questions on intention to change behavior regarding clients that are having pain were answered on a 7-point rating scale where 1 indicated lack of importance, disagreement, or unlikeliness and 7 indicated high importance, agreement, or likeliness. The survey section aimed to assess intention to change behavior was only completed by direct care providers.

### Analysis

The study was designed as an interrupted time series where the RAI-HC data were available for 22 months (partitioned into 6 pre-intervention months, 10 intervention months, and 6 post-intervention months). A segmented regression analysis was employed to statistically evaluate the magnitude of the effect of the feedback reports on the quality indicators of interest. RAI-HC data available from each of the 22 months were grouped so that the pre-intervention period had 6 monthly time points (December 1, 2010, to May 31, 2011), the intervention period had 10 monthly time points (June 1, 2011, to March 31, 2012), and the post-intervention period had 6 monthly time points (April 1, 2012, to September 30, 2012) for interrupted time series. Each quality indicator was assessed before, during, and after the feedback report intervention for changes in level and slope in specific time series. A predicted regression line was fitted to each segment for quality indicators. The relationship between time and the quality indicator within each segment in the model was assumed to be linear. The specified linear regression model is as follows:

$$ {Y}_t={b}_0+{b}_1*{\mathrm{time}}_t+{b}_2*{\left(\mathrm{feedback}\right)}_t + {b}_3*{\left(\mathrm{time}\ \mathrm{after}\ \mathrm{feedback}\right)}_t+{b}_4*{\left(\mathrm{without}\ \mathrm{feedback}\right)}_t + {b}_5*{\left(\mathrm{time}\ \mathrm{after}\ \mathrm{without}\ \mathrm{feedback}\right)}_t+{e}_t $$

where

- *Y*_*t*_ is the percentage of clients with the quality indicator in month *t*
- time is the time in months at time *t* from the start of the observation period, it ranges from 1 to 22 months
- feedback is an indicator for time *t* occurring before (feedback = 0) or after (feedback = 1) the feedback report, which was implemented in June 2011 in the series
- time after feedback is the number of months after the intervention at time *t*, coded 0 before the feedback report and (time − 6) after the feedback report;
- without feedback is an indicator for time *t* occurring before (without feedback = 0) or after (without feedback = 1) without the feedback report, which was after March 2012 in the time series
- time after without feedback is the number of months after the intervention at time *t*, coded 0 before the end of the feedback report and (time − 16) after the end of the feedback report
- *e*_*t*_ is the error term at time *t* that represents the unexplained random variation in the model

In this model, regression parameters are estimated as follows:

- *b*_0_, percentage of clients with the health outcome at baseline
- *b*_1_, trend prior to the feedback report (baseline trend)
- *b*_2_, change in level immediately after the first feedback report
- *b*_3_, change in trend after the feedback report distribution
- *b*_4_, change in level immediately after the last feedback report
- *b*_5_, change in trend after the end of the feedback report distribution

Serial autocorrelation of the error terms was tested in the regression model using the Durbin-Watson (DW) statistic. Theoretically, possible values of the DW statistic range from 0 to 4. Observations close to midpoint of 2.00 indicate no serious autocorrelation. If serial correlation was detected in the data, correction would be made using the Prais-Winsten estimator.

Data on client demographics was collected at four points during the study: December 2010, June 2011, March 2012, and September 2012. Statistical analyses were conducted using STATA version 12 (StataCorp, College Station, TX), and statistical significance was defined at a level of *α* = 0.05.

## Results

### Resident characteristics

Over the entire study period, data from 548 participants are reported. The average age of clients was similar at study baseline (December 2010), study completion (September 2012), feedback report initiation (June 2011), and conclusion (March 2012). Home care clients were predominantly female (Table 1).

**Table 1:** Client demographics at study initiation, initiation and conclusion of feedback report period, and study conclusion

Demographic characteristics	Study initiation (December 2010, *n* = 54)		Intervention initiation (June 2011, *n* = 141)		Intervention conclusion (March 2012, *n* = 225)		Study conclusion (September 2012, *n* = 128)	
Age (in years)								
Mean	82.2		78.2		77.5		77.8	
Standard deviation	10.1		14.2		16.5		13.3	
Sex	No.	%	No.	%	No.	%	No.	%
Male	16	29.6	56	39.7	77	34.2	54	42.2
Female	38	70.4	85	60.3	148	65.8	74	57.8

### Autocorrelation

Each quality indicator in the model was estimated using simple linear regression with an additional test for first-order autocorrelation. Serial autocorrelation was only present in pain (DW statistic = 1.45), and so Prais-Winsten estimator was used to correct for this quality indicator.

### Segmented regression analysis

**Fig. 1:**
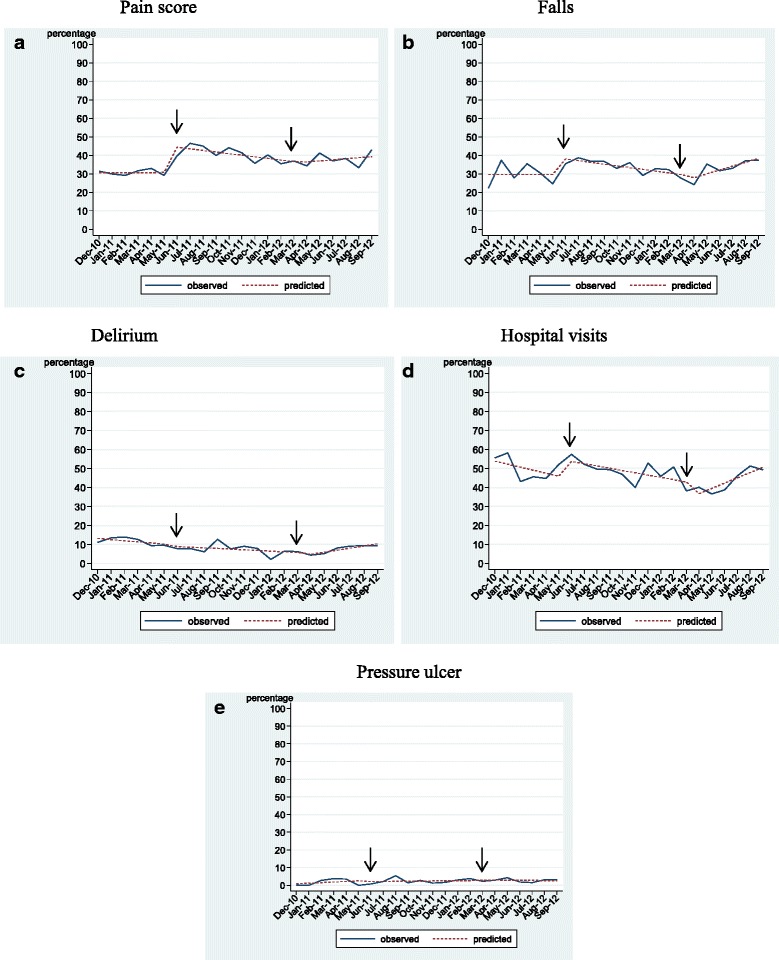
**a**–**e** Time series quality indicator outcomes

Figure 1a–e depicts the five time series quality indicator outcomes. Findings of the segmented regression analysis are summarized in Table 2. Analyses showed a mixed pattern of results across the four quality indicator outcomes of interest. The implementation of feedback reporting was not associated with the baseline trend uniformly across quality indicators. As well, the change in the trend across quality indicators after feedback report implementation were not uniformly associated with the implementation of feedback reporting indicating that audit with feedback in home care may only impact select quality indicators.

**Table 2:** Parameter estimates for percentage of clients with pain, falls, delirium, hospital visits, and pressure ulcer

	Coefficient estimate	Standard error	*t-*statistic	*p* value
Pain score				
*b*_0_	30.69	2.72	11.27	<0.01
*b*_1_	0.01	0.70	0.01	0.99
*b*_2_	14.80	2.91	5.08	<0.01
*b*_3_	−0.89	0.77	−1.16	0.26
*b*_4_	−0.87	3.22	−0.27	0.79
*b*_5_	1.49	0.77	1.93	0.07
Falls				
*b*_0_	29.70	3.77	7.87	<0.01
*b*_1_	−0.01	0.97	−0.01	0.99
*b*_2_	9.35	4.03	2.32	0.03
*b*_3_	−0.93	1.07	−0.87	0.40
*b*_4_	−3.79	4.46	−0.85	0.41
*b*_5_	3.03	1.07	2.84	0.01
Delirium				
*b*_0_	13.77	1.94	7.10	<0.01
*b*_1_	−0.60	0.50	−1.20	0.25
*b*_2_	−0.93	2.07	−0.45	0.66
*b*_3_	0.25	0.55	0.46	0.65
*b*_4_	−2.12	2.29	−0.92	0.37
*b*_5_	1.45	0.55	2.65	0.02
Hospital visit				
*b*_0_	55.41	4.59	12.08	<0.01
*b*_1_	−1.59	1.18	−1.35	0.20
*b*_2_	9.00	4.90	1.83	0.09
*b*_3_	0.39	1.30	0.30	0.77
*b*_4_	−8.92	5.42	−1.65	0.12
*b*_5_	3.97	1.30	3.06	0.01
Pressure ulcer				
*b*_0_	0.52	1.42	0.37	0.72
*b*_1_	0.33	0.36	0.91	0.38
*b*_2_	−0.52	1.52	−0.34	0.74
*b*_3_	−0.26	0.40	−0.64	0.53
*b*_4_	0.30	1.68	0.18	0.86
*b*_5_	−0.16	0.36	−0.43	0.67

*b*_*0*_ percentage of clients with the quality indicator (health outcome) at baseline, *b*_*1*_ trend of quality indicator prior to the feedback report (baseline trend), *b*_*2*_ change in level of quality indicator immediately after the first feedback report, *b*_*3*_ change in trend of quality indicator after the feedback report distribution; *b*_*4*_ change in level of quality indicator immediately after the last feedback report, *b*_*5*_ change in trend of quality indicator after the end of the feedback report distribution.

### Pain

The number of clients reporting pain ranged from 30 to 50% between December, 2010, and September, 2012. The coefficient estimate for the change in level of the proportion of clients reporting high pain scores at initiation of the feedback report (*b*_2_) indicates a significant increase in reporting of pain. The negative value of the regression coefficient estimate for the feedback report phase (*b*_3_) of the study indicates a decrease in the proportion of clients with high pain scores relative to baseline trend, but the measure is not significant. There were no other statistically significant coefficient estimates for pain (Fig. 1a).

### Falls

The number of clients reporting a fall ranged from 20 to 40% between December, 2010, and September, 2012. As in the case of pain, there was a significant increase in the coefficient estimate for the change in level of the proportion of clients who had a fall at feedback report initiation (*b*_2_). The negative value of the regression coefficient estimate for the feedback report period of the study (*b*_3_) indicates a decrease (not significant) in the proportion of clients reporting a fall relative to baseline trend. In the post-intervention period, the regression coefficient estimate (*b*_5_) indicates a statistically significant increase for falls relative to the feedback report period. There were no other statistically significant coefficient estimates for pain (Fig. 1b).

### Delirium

The number of clients reporting delirium ranged from 4 to 14% over the observation period. There were no significant changes in the percentage of clients reporting delirium in the pre-intervention or feedback reporting periods, and there were no changes in the level of feedback reporting at the initiation or conclusion of feedback reporting. However, the positive value of the regression coefficient estimate for the post-feedback report period of the study (*b*_5_) indicates a significant increase in the proportion of clients reporting delirium relative to the 10-month intervention period (Fig. 1c).

### Hospital visits

The number of clients reporting hospital visits ranged from 35 to 60% over the observation period. Though the negative slope of the regression line for the feedback reporting period indicates a decrease in hospital visits, the value of the regression coefficient estimate for this period (*b*_3_) suggests no change in reporting of hospital visits relative to the baseline trend. However, there was a significant increase in the proportion of clients reporting hospital visits in the post-feedback report period (*b*_5_) relative to the 10-month feedback report period. There were no other statistically significant coefficient estimates for hospital visits (Fig. 1d).

### Pressure ulcer

The percentage of clients reporting pressure ulcer was low throughout the 22-month observation period and did not exceed 6%. There was no statistically significant change in the trend of the percentage of clients reporting a pressure ulcer before, during, or after the feedback reporting phases or in the level of pressure ulcer reported at implementation or conclusion of feedback reporting (Fig. 1e).

### Process evaluation

There were a total of 300 responses to feedback report surveys in the study (Table 3). Response rate to feedback report survey ranged from 46% for the first survey (distributed after the first feedback report) to 56% for the third survey (distributed after the third feedback report). Several types of care providers completed the surveys: healthcare aides constituted the majority of respondents to the first survey (25%), case managers constituted the majority of respondents to the second (39%) and third surveys (38%), and registered nurses were the most abundant respondents to the fourth survey (33%). Overall the four survey periods, 87% of respondents indicated they read more than half of the report, 86% indicated understanding more than half of the report, 43% found the report useful, 38% discussed the report with another staff member, and 35% found the report useful to make changes in the way they take care of clients. One third of the respondents indicated that the feedback reports were discussed at staff meetings. Based on the feedback reports, the respondents who indicated that they desired changes made in the way clients are taken care of cited primarily that the information is useful to other care providers (not necessarily themselves) and changes should be made in the way clients are assessed and assisted in daily living, the policies that affect clients, and the daily schedule of clients. The mean respondent scores (±standard deviation) for intention to assess of monitor clients’ level of pain during each shift for surveys 1, 2, 3, and 4 were 4.58 (±1.72), 4.95 (±1.83), 5.47 (±1.44), and 5.17 (±1.72), respectively on the 7-point rating scale.

**Table 3:** Process evaluation outcomes of an audit with feedback in home care

	June 2011		September 2011		December 2011		March 2012		Overall	
	(*n* = 63)		(*n* = 74)		(*n* = 87)		(*n* = 75)			
	No.	%	No.	%	No.	%	No.	%	No.	%
Position title										
Care/case manager	12	19.0	29	39.2	33	37.9	15	20.0	89	29.8
Registered nurse	12	19.0	18	24.3	20	23.0	25	33.3	75	25.1
Licensed practical nurse	6	9.5	4	5.4	7	8.0	4	5.3	21	7.0
Healthcare aide/personal care attendant	16	25.4	14	18.9	14	16.1	19	25.3	63	21.1
Allied health professionals	12	19.0	6	8.1	6	6.9	3	4.0	27	9.0
Manager/team leader	5	7.9	2	2.7	5	5.8	4	5.3	16	5.4
Others	–	–	1	1.4	2	2.3	5	6.7	8	2.7
Provider responses to feedback reports										
Read more than half of the report	57	90.5	68	91.9	70	84.3	61	81.3	256	86.8
Understood more than half of the repot	54	87.1	65	86.7	73	85.9	63	84.0	255	85.9
Found the report useful overall	28	44.4	32	42.7	38	44.2	29	39.7	127	42.8
Discussed the report with another staff member	19	30.2	26	34.7	32	37.7	35	47.3	112	37.3
Found the report useful to make changes in the way they take care of clients	25	40.3	24	32.0	34	40.5	20	26.7	103	34.8

## Discussion

This study reports on an audit with feedback intervention delivered over 10 months across seven home care offices. It was expected that the percentage of clients who experienced high pain, falls, delirium, or hospital visits would decrease during the intervention phase. It was also expected that the decline in quality indicators would be sustained after the end of the feedback report distribution. Feedback reporting showed that there were decreases in pain, fall, delirium, and hospital visits over the intervention period but that there was no difference in the trend for reporting quality indicators in the intervention period relative to the pre-intervention period.

Contrary to expectation, the decreases in pain, falls, delirium, and hospital visits observed during the intervention period were not sustained beyond the 6-month post-intervention period. The change in trend for pain, falls, delirium, and hospital visits all increased in the post-feedback report period relative to the intervention period. These data suggest that audit with feedback intervention may need to be an ongoing process to sustain potential improvement in pain, falls, delirium, and hospital visits or that the intervention may require inclusion of additional elements to maintain or continue to improve intervention-related increases in the quality indicators of interest.

According to this study, the initiation of feedback reporting was associated with an increased level in the proportion of clients reporting pain, falls, and possibly hospital visits, but not with delirium. On the other hand, there were no changes in the levels of pain, falls, delirium, or hospital visits reported immediately following intervention (after distribution of final feedback report).

Although there was large month-to-month variability in fall and hospital visits reported in the pre-feedback period, there was no significant linear trend for any of the four quality indicators of interest over the 6 months prior to intervention. Finally, pressure ulcer served as an appropriate reference quality indicator as there was no change in reporting on level or trend of this outcome among any of the study periods.

### Facilitators and barriers to audit with feedback in home and long-term care settings

Some of the differences in outcomes observed between the present study in home care and the previously published study in long-term care may be a result of the frequency of report distribution, the source of the feedback, and the perceived sign (whether positive, neutral, or negative) of the information in the report. Feedback reporting occurred monthly in the DICE-LTC study and quarterly in the DICE-HC study. Due to the small magnitude of effects observed, the results of both studies do not provide evidence as to the optimal frequency of feedback reporting in continuing care. Furthermore, feedback report delivery was done in person in the DICE-LTC study, and some feedback reports were delivered electronically in the DICE-HC study. Electronic distribution can reach a great proportion of staff in a timely manner, though electronic distribution may also be ignored without verbal notification or filtered from view in email. In-person distribution relies heavily on a “site champion” to engage and make staff aware of feedback reports. This is especially important as staff in home care working remotely may not come to respective home care offices frequently to pick up feedback reports in mail.

It was proposed that the post-feedback report survey may have unintentionally served as a co-intervention to feedback reports unintentionally causing the decrease in reports of high pain scores in long-term care. Since the audit with feedback study in home care also involved post-feedback report surveys and actually demonstrated an increase in reports of high pain scores at the beginning of the intervention period, it is unlikely that the post-feedback report surveys acted as a co-intervention tool in LTC or in the home care study reported on here.

### Process evaluation

More than 90% of the staff read more than half of the first two feedback reports. Only about 80% of the staff read more than half of the final feedback report. The decrease in feedback report reading over time may indicate that the staff felt that report distribution intervals too frequent to notice differences or changes in the trend of quality indicators. Alternatively, positive or negative trends of quality indicators in feedback report may have influenced the desire of the staff to continue reading feedback reports over time. The staff in home care discussed reports with others less often than the staff in LTC. This is because home care staff may not have the same opportunity to discuss feedback reports with peers and colleagues that staff in LTC had due to the nature of the work environment. Providers in home care are usually based remotely in the community where interaction with colleagues cannot occur very often.

More than 50% of the respondents in the DICE-LTC study were healthcare aides compared to less than 25% in DICE-HC. The range of abilities and role boundaries of healthcare aides differ from that of other professions. There is variable education and training for different care provider professions (e.g., licensed practical nurse, healthcare aide, social worker). As such, a recent study of audit with feedback in home care on pain and falls in Ontario, Canada, suggests that feedback reporting may benefit from being catered to specific provider groups [27]. The findings from the DICE-HC study support this notion as almost half of the respondents indicated that they found reports useful overall, but only about one third of the respondents found the report useful to make changes to the way they cared for clients. This indicates that relevant information regarding client care may be present in the feedback report, but the care provider reading the report does not necessarily believe that they can positively influence the specific client outcome. This may be because the care provider reading the report believes that the activities performed within the role, competencies, and scope of practice of their profession do not have great influence on a specific quality indicator. Improvement in quality indicators are often achieved as a result of collaborative care planning in teams, not solely through individual efforts. It would be of interest to explore aspects of interprofessional collaboration and team climate in home care as it relates client outcomes in future study in order to discern which care professionals and how specific care professionals believe they can impact quality indicators.

### Limitations

Best practice recommendations made from the present study may be limited by the quasi-experimental design. The degree on RAI-HC implementation across provincial home care offices did not allow for inclusion of a contemporaneous control wherein the measurement of quality indicators could be obtained from home care offices not undergoing audit with feedback intervention. Interrupted time series study designs may be restricted by the number of times at which the dependent variable is assessed. In order to satisfy the requirements for the use of an interrupted time series design, quarter-annual report data were instead analyzed monthly so that three times as many time segments were available for analyses. It is possible that in addition to changes in care planning behavior, the intervention prompted changes to the attention given to specific parameters in administering the RAI-HC. It was a challenge to deliver feedback reports electronically as email notifications can often be ignored and the process requires buy-in from a site champion; as such, the collective study data may be more representative of the Edmonton, North, and Central Zones where feedback reports were distributed in person. Irrespective of perceived study limitations, this is one of the longest and most intensive studies of an audit with feedback intervention conducted in the home care setting.

## Conclusions

Routinely collected data when used over time for feedback is a feasible approach for a quality improvement initiative. There is a need to devise strategies for implementation, effective delivery, and follow-up of the intervention in addition to the resources required to achieve beneficial outcomes, for example, more resources for distribution and real-time discussion of results. Pain, falls, delirium, and hospital visits increased in the post-feedback report period after decreasing during the intervention period. Thus, this study supports the ongoing use of audit with feedback using client data from the RAI-HC so that care givers can continue to provide client-centered service for optimal outcomes in the home care setting.

## Additional file

Additional file 1: DICE Project Feedback Report, December 2011

## Abbreviations

DICE-HC: Data for Improvement and Clinical Excellence – Home Care
DICE-LTC: Data for Improvement and Clinical Excellence – Long-Term Care
LTC: Long-term care
RAI-HC: Resident assessment instrument – home care
