# Supplementary material for: Data for Improvement and Clinical Excellence: a report of an interrupted time series trial of feedback in home care
Source: Implement Sci. 2017 May 18;12:66. doi: 10.1186/s13012-017-0600-1 (PMC5437696; doi:10.1186/s13012-017-0600-1)
Supplement: Additional file 1: — DICE Project Feedback Report, December 2011 [file 13012_2017_600_MOESM1_ESM.pdf]

Additional File 1

Site: CTFHL

DICE Project
Feedback Report, December 2011

➢ PAIN

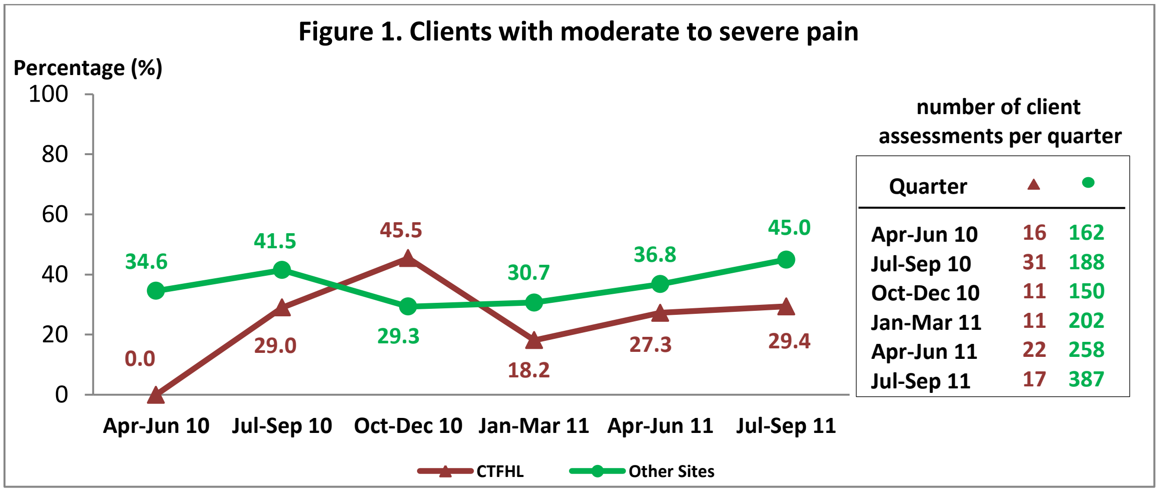

- The information in Figure 1 is from items k4a and k4b of the RAI-HC
- These two items are used to measure the proportion of clients with moderate to severe pain
- In the July-September 2011 quarter, there was almost no change in the proportion of clients in CTFHL with moderate to severe pain; it was lower than the other sites

➢ FALLS

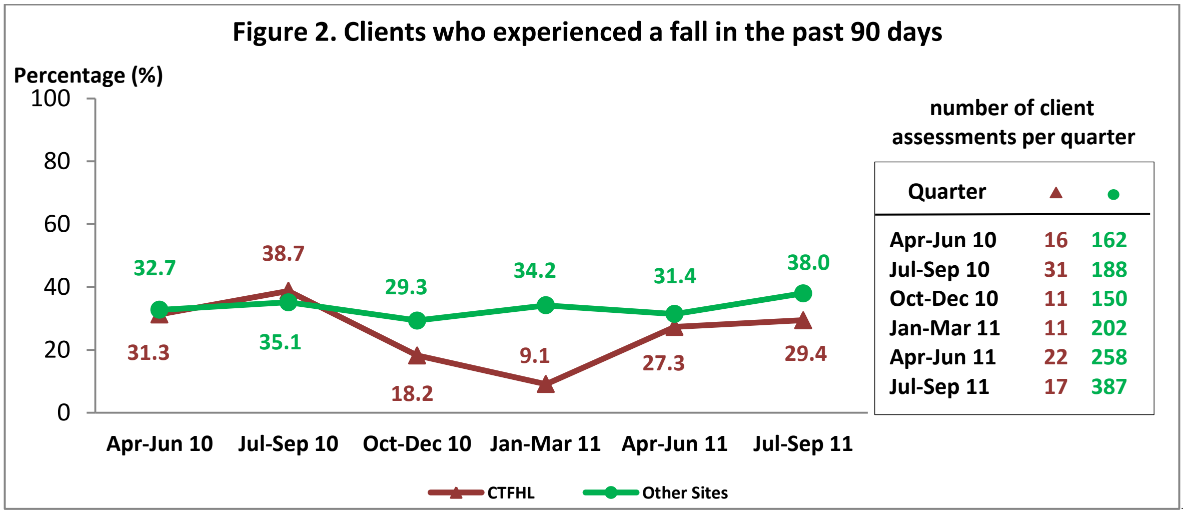

- The information in Figure 2 is from item k5 of the RAI-HC
- This item is used to measure the proportion of clients who experienced a fall in the past 90 days
- In the July-September 2011 quarter, there was almost no change in the proportion of clients in CTFHL who experienced a fall in the past 90 days; it was lower than the other sites

➢ DELIRIUM

Figure 3. Clients with indications of delirium

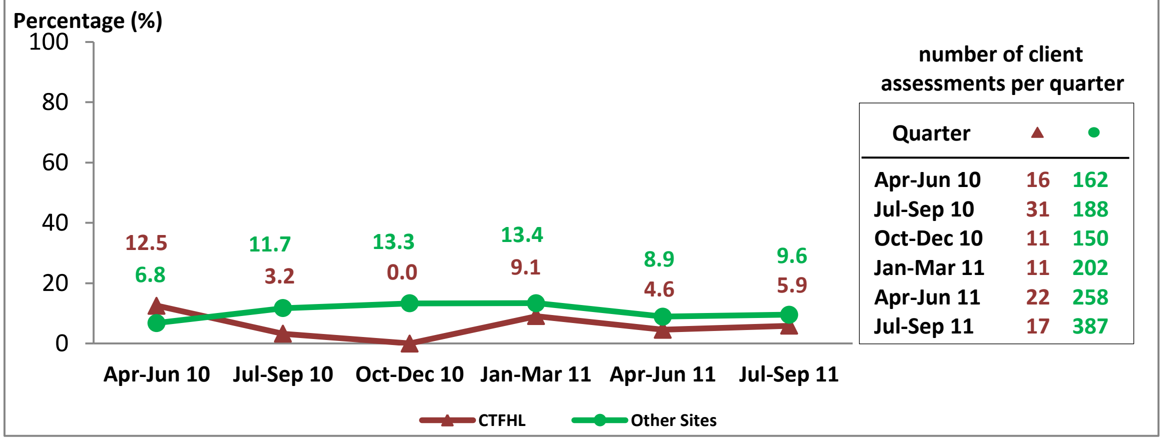

- The information in Figure 3 is from items b3a and b3b of the RAI-HC
- These two items are used to measure the proportion of clients with indications of delirium
- In the July-September 2011 quarter, there was almost no change in the proportion of clients in CTFHL who had indications of delirium; it was about the same as the other sites

➢ HOSPITALIZATION

Figure 4. Clients who have been hospitalized, visited hospital emergency department or received emergent care

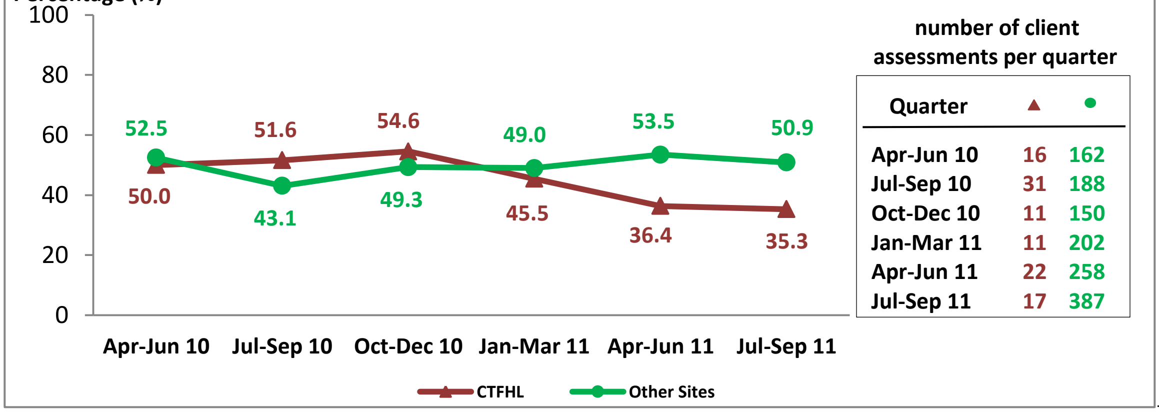

- The information in Figure 4 is from items p4a, p4b and p4c of the RAI-HC
- These three items are used to measure the proportion of clients who have been hospitalized, visited hospital emergency department or received emergent care
- In the July-September 2011 quarter, there was almost no change in the proportion of clients in CTFHL who have been hospitalized, visited hospital emergency department or received emergent care; it was lower than the other sites

SOURCE: *Data for Improvement and Clinical Excellence (DICE) Project, Faculty of Nursing, University of Alberta*
